# Subnuclear localisation is associated with gene expression more than parental origin at the imprinted *Dlk1-Dio3* locus

**DOI:** 10.1371/journal.pgen.1010186

**Published:** 2022-04-28

**Authors:** Rahia Mashoodh, Lisa C. Hülsmann, Frances L. Dearden, Nozomi Takahashi, Carol Edwards, Anne C. Ferguson-Smith

**Affiliations:** 1 Department of Zoology, University of Cambridge, Cambridge, United Kingdom; 2 Department of Genetics, University of Cambridge, Cambridge, United Kingdom; University of Pennsylvania, UNITED STATES

## Abstract

At interphase, de-condensed chromosomes have a non-random three-dimensional architecture within the nucleus, however, little is known about the extent to which nuclear organisation might influence expression or *vice versa*. Here, using imprinting as a model, we use 3D RNA- and DNA-fluorescence-in-situ-hybridisation in normal and mutant mouse embryonic stem cell lines to assess the relationship between imprinting control, gene expression and allelic distance from the nuclear periphery. We compared the two parentally inherited imprinted domains at the *Dlk1-Dio3* domain and find a small but reproducible trend for the maternally inherited domain to be further away from the periphery however we did not observe an enrichment of inactive alleles in the immediate vicinity of the nuclear envelope. Using Zfp57KO ES cells, which harbour a paternal to maternal epigenotype switch, we observe that expressed alleles are significantly further away from the nuclear periphery.

However, within individual nuclei, alleles closer to the periphery are equally likely to be expressed as those further away. In other words, absolute position does not predict expression. Taken together, this suggests that whilst stochastic activation can cause subtle shifts in localisation for this locus, there is no dramatic relocation of alleles upon gene activation. Our results suggest that transcriptional activity, rather than the parent-of-origin, defines subnuclear localisation at an endogenous imprinted domain.

## Author summary

Genomic imprinting is an epigenetically regulated process that results in the preferential expression of a subset of developmentally regulated genes from either the maternally or the paternally inherited chromosomes. We have used imprinted genes as a model system to investigate the relationship between the parental origin of the chromosomes, the localisation of genes within the cell nucleus and their active expression. By assessing the location of the *Dlk1-Dio3* region and the expression of the *Gtl2/Meg3* gene within it, we find that there is a small preference for the paternal chromosome to be closer to the periphery but a significant correlation between transcription and distance to the edge of the nucleus for the *Gtl2/Meg3* gene. Furthermore, a chromosome nearer the periphery is just as likely to express the gene as the chromosome further away. These findings suggest that the parental origin of the chromosome may not be as important as the transcription of the gene in defining the position of the imprinted region within the nucleus.

## Introduction

The spatial organization of chromosomes in the interphase nucleus is non-random and involves 3D interactions on chromatin as well as interactions with various nuclear domains, the nuclear envelope being the best characterized [1]. While the bulk of chromosomes is arranged as relatively compact and spatially defined chromosome territories [2], open chromatin between these contains transcription factories where active genes from different genomic loci co-localize at times of transcription [3]. Recently, there has been much interest in understanding ways in which the 3D localisation of chromatin can influence or be influenced by gene expression [4,5]. While chromatin organization into chromosome territories, topologically associated domains, and loops have complex effects on transcriptional regulation, interactions with the nuclear envelope are generally seen as transcriptionally repressive [1]. Lamina associated domains (LADs) have been characterized as gene poor, transcriptionally inactive regions [6], even though these repressed regions have been shown to be dynamic in dividing cells and are therefore not always lamina-associated in every cell within a population [7]. A notable exception to the repressive environment at the nuclear periphery is represented by nuclear pore complexes, where chromatin-facing nucleoporins have been implicated in transcriptional activation rather than repression in yeast [8]. However, in Drosophila, nucleoporins appear to have this activating effect mainly in the nuclear interior [9–11]. In line with the general notion that the nuclear periphery acts as a repressive environment, some though not all individual genomic loci have been shown to become transcriptionally repressed after artificial targeting to the nuclear lamina [12–17]. These findings suggest a role for subnuclear position in gene regulation, however, the extent and importance of non-random localisation is poorly understood.

**Fig 1:**
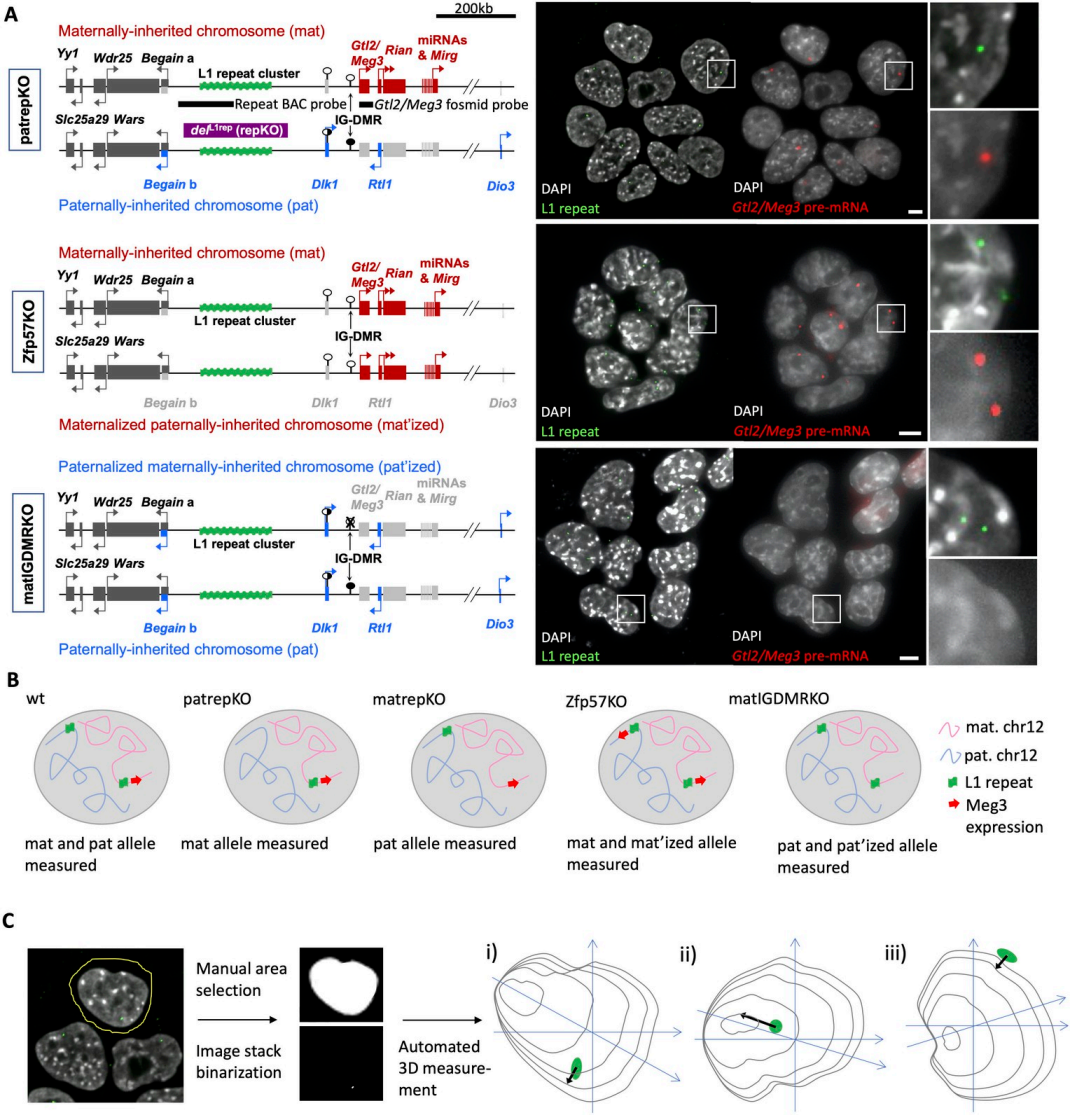
Experimental design. (A) A 260kb deletion encompassing the LINE1 cluster in the *Dlk1-Dio3* imprinted region was used to mark the parental origin of alleles. ES cells derived from mutant mice were used that mimic a uniparental origin of the *Dlk1-Dio3* region. ES cells with a homozygous deletion of the chromatin modifier Zfp57 show maternal expression patterns at the paternally inherited chromosome while ES cells with a maternally inherited deletion of the IGDMR show paternal expression patterns from the maternally inherited chromosome. The FISH images of ES cells, where the maternal L1 cluster was used for 3D measurements and *Gtl2/Meg3* expression was assessed in parallel. As predicted, many Zfp57KO ES cells showed biallelic expression of maternally expressed *Gtl2/Meg3*, while matIGDMRKO ESC showed no *Gtl2/Meg3* expression. Chromosome maps are derived from Soares et al. 2018 [21]. The FISH images represent maximum projections of 20–30 central z planes of acquired image stacks. Blow-ups of framed regions are shown on the right. Scale bars represent 5μm. (B) Schematic of nuclei with the expected combined DNA and RNA FISH results for all genotypes. (C) Automated, unbiased, distance measurements using the Fiji imaging package. Each nucleus is manually selected from the FISH image stack and both channels are binarized and added to Fiji’s 3D manager to automatically measure the shortest distance from the centre of the FISH signal to the chromatin border in 3D. The shortest distance might be oriented more in the xy axis (i) or more in the z axis (ii) of the image stack. Occasionally, peripheral FISH signals were just outside of the chromatin representation (iii) and therefore measured as negative distances (See Methods).

The *Dlk1-Dio3* imprinted domain on mouse chromosome 12 is well characterized and is a valuable model for comparative analysis of gene regulatory mechanisms. Imprinted genomic regions contain genes that are preferentially expressed from either the maternally or paternally inherited chromosome, but that also are subject to the same regulated and stochastic transcriptional mechanisms that govern the expression of other genes. Imprinted genes are therefore regulated by both germline-derived parental-origin-specific epigenetic mechanisms and the transcriptional milieu of a particular cell type [18,19]. *Dlk1-Dio3* imprinting (Fig 1A) is regulated by an intergenic differentially methylated region (IGDMR) and contains a number of paternally expressed protein-coding genes as well as maternally expressed non-coding RNAs. Recent evidence has explored the relationship between the parental origin of this imprinted domain and its subnuclear locations. Kota and colleagues identified differential localisation of the *Gtl2/Meg3* gene in a mouse embryonal stem (ES) cell line with the non-expressed paternal copy being closer to the nuclear periphery than the expressed maternal allele [20]. Furthermore, a LINE1 (L1) repeat cluster located between *Begain* and *Dlk1* within the cluster (Fig 1A) was described to represent a facultative LAD in mouse ES cell-derived neural stem cells [21,22]. Although this suggests that the L1 repeat might have an inhibiting effect on local gene expression via lamina tethering, deletion of the repeat cluster itself did not lead to increased expression of neighbouring genes [21]. Here, we use extensive 3D RNA-DNA fluorescence-in-situ-hybridisation (FISH) in multiple normal and mutant mouse ES cell lines and show that gene expression state rather than the parental origin of alleles is associated with differences in intranuclear localisation, and suggest that juxtaposition to the nuclear envelope appears to play a minor role, if any, in gene regulation at this endogenous locus.

## Results

### Differential intranuclear distribution of parental alleles of the *Dlk1-Dio3* region

In order to analyse the intranuclear localisation of alleles of the *Dlk1-Dio3* region, while knowing their parental origin, we generated ES cell lines derived from *del*^L1rep^ maternal and paternal heterozygous mice [21]. We used only male ES cell lines for this study since it has been previously shown that imprinting status is more stable in male ES cells compared to female ES cells [23]. These cells carry a deletion between the genes *Begain* and *Dlk1*, which includes a 170kb L1 repeat array (Figs 1A and S1) and have a normal epigenotype. Using a DNA FISH probe specific to the L1 repeat array, we used the deletion to distinguish the two parental chromosomes in 3D DNA FISH experiments, independent of gene expression. Importantly, we quantified the behaviour of WT chromosomes not carrying the deletion. For each cell we measured the 3D distance of the DNA FISH probe to the nuclear periphery defined by the edge of DAPI staining of chromatin in an automated way (Fig 1C). To ensure that DAPI staining faithfully captured the position of the lamina, using a separate set of ES cells (n = 84), we compared edge measurements taken by DAPI staining compared to staining of the lamin in the same cells. Whilst lamin staining results in two visible borders (an outer and an inner) both measurements were highly correlated, suggesting that DAPI provides a reasonable estimate of the relative distance relationships between cell lines (t = 94.309, p<2e-16, estimate (slope) = 0.993 and t = 138.12, p<2e-16, estimate (slope) = 0.996 for inner and outer lamin border, respectively; S2 Fig). Therefore, the DAPI edge measurement was used as a read-out for the intranuclear position of the *Dlk-Dio3* imprinted region, which lies within a single TAD in ES cells (S1 Fig) [24,25]. In parallel, we used nascent RNA FISH to obtain the *Gtl2/Meg3* expression state (expressed or non-expressed) for each allele.

**Fig 2:**
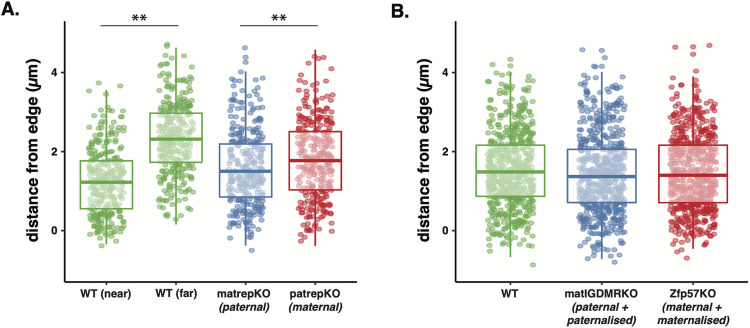
Distribution of alleles by parental origin. (A) Paternal alleles (matrepKO–blue datapoints) of the *Dlk1-Dio3* are significantly closer to the nuclear periphery than maternal alleles (patrepKO–red datapoints), as has been reported previously. This difference is less pronounced than the natural difference between the two alleles (near and far) within a WT cell (**p<0.*01*). (B) There were no differences in the distance from the periphery between paternal and paternalized alleles (blue datapoints) from matIGDMRKO ES cells or maternal and maternalized alleles (red datapoints) from Zfp57KO ES cells from WT cells.

We took DNA FISH probe distance measurements in three biological replicates each of heterozygous ES cells derived from maternal inheritance of *del*^L1rep^ (matrepKO), paternal inheritance of *del*^L1rep^ (patrepKO) and WT ES cells, this way comparing paternally inherited alleles, maternally inherited alleles and the mixture of both as a control. Using a multiple least-squares linear regression model, we find that paternal alleles are significantly closer to the nuclear periphery compared to maternal alleles after controlling for nuclear volume (t = 2.946, p = 0.00334, estimate (slope) = 0.22 μm; Fig 2A). Given that WT cells contained both alleles, we decided to characterise the relationship between the two alleles to understand their natural distribution relative to each other within these cells. In WT cells, one allele was always closer to the nuclear border than the other, and there was a significant difference between the two alleles (t = 22.80, p<2e-16, estimate (slope) = 1.08 μm; Fig 2A). This is consistent with a second set of WT cells that were used in this study (WT2; t = 22.59, p<2e-16, estimate (slope) = 0.99 μm). While the parental origin of the WT alleles was unknown, we assume that this difference might, at least in part, reflect the tendency for the paternal allele to be biased towards the periphery, as demonstrated in previous reports [20]. However, in the KO cells, where the accuracy of parental origin is ensured, the average difference in distance between the maternal (*M* = 1.81 μm, *SD* = 1.00 μm) and the paternal allele (*M* = 1.58 μm, *SD* = 0.954 μm) was unexpectedly small (absolute raw mean difference of ~230nm). Previous work from Kota and colleagues had found differences of more than a micrometer [20], a difference that is consistent to that between the WT near and far alleles regardless of parental origin, but not the maternal and paternal alleles in this study (Fig 2A). This small difference that we observed in KO cells was not due to variability in replicates as replicates were not significantly different from one another (S3 Fig), but suggested that the parental origin effect we observed might be of limited functional significance. We, therefore, developed a genetic approach to compare the two parental chromosomes in a more functional context.

### Epigenotype switching does not shift localisation

To address the relationship between parent of origin and function more overtly we utilized genetic models exhibiting an epigenotype switch that reversed the imprints on either the maternal or the paternal chromosome. We use the term ‘epigenotype’ to refer to the parental- origin specific epigenetic marks (both histone modifications and DNA methylation) that distinguish the two parental chromosomes. For example, the paternal and maternal IG-DMR are enriched with H3K9me3 and H3K4me3/H3K27ac, respectively [19,26–28]. The maternally inherited *Gtl2* promoter also exhibits substantial parental origin specific differential DNA methylation—the paternal allele is hypermethylated, the maternal allele is hypomethylated [19,28].

Zfp57KO ES cells carry a null mutation in the chromatin modifier ZFP57, which is important for imprint maintenance and, in the case of the *Dlk1-Dio3* region, results in a paternal to maternal epigenotype switch causing a maternal expression pattern from the paternally inherited chromosome (Figs 1A and 1B and S4) [26,29] Conversely, matIGDMRKO ES cells are derived from blastocysts that have a maternally inherited deletion of the IGDMR, resulting in a maternal to paternal epigenotype switch and expression of the paternally expressed imprinted genes from the maternally inherited chromosome [30]. Therefore, in matIGDMRKO and Zfp57KO lines, both copies of the imprinted domain now behave (*i*.*e*., are expressed) as if they were paternal or maternal copies, respectively. For this experiment, we generated additional matched WT control ES cells (WT2) using WT littermates of KO blastocyts as before (see Methods). Using three biological replicates for each mutant and control line, we found that there was no significant difference in the subnuclear localisation of mutant chromosomes compared to WT chromosomes between cell types (t = 0.143, p = n.s; Fig 2B). This was true, even after effects of nuclear volume were taken into account. These findings indicate that maternalising the paternal domain and paternalising the maternal domain, do not result in the expected shift in localisation and therefore indicate that epigenotype is not determining the localisation. However, these data do not consider the current transcriptional state of imprinted loci in these models.

### Stochastic or developmental repression of the maternally expressed *Gtl2/Meg3* gene correlates with a shift of alleles towards the nuclear periphery in ES cells

**Fig 3:**
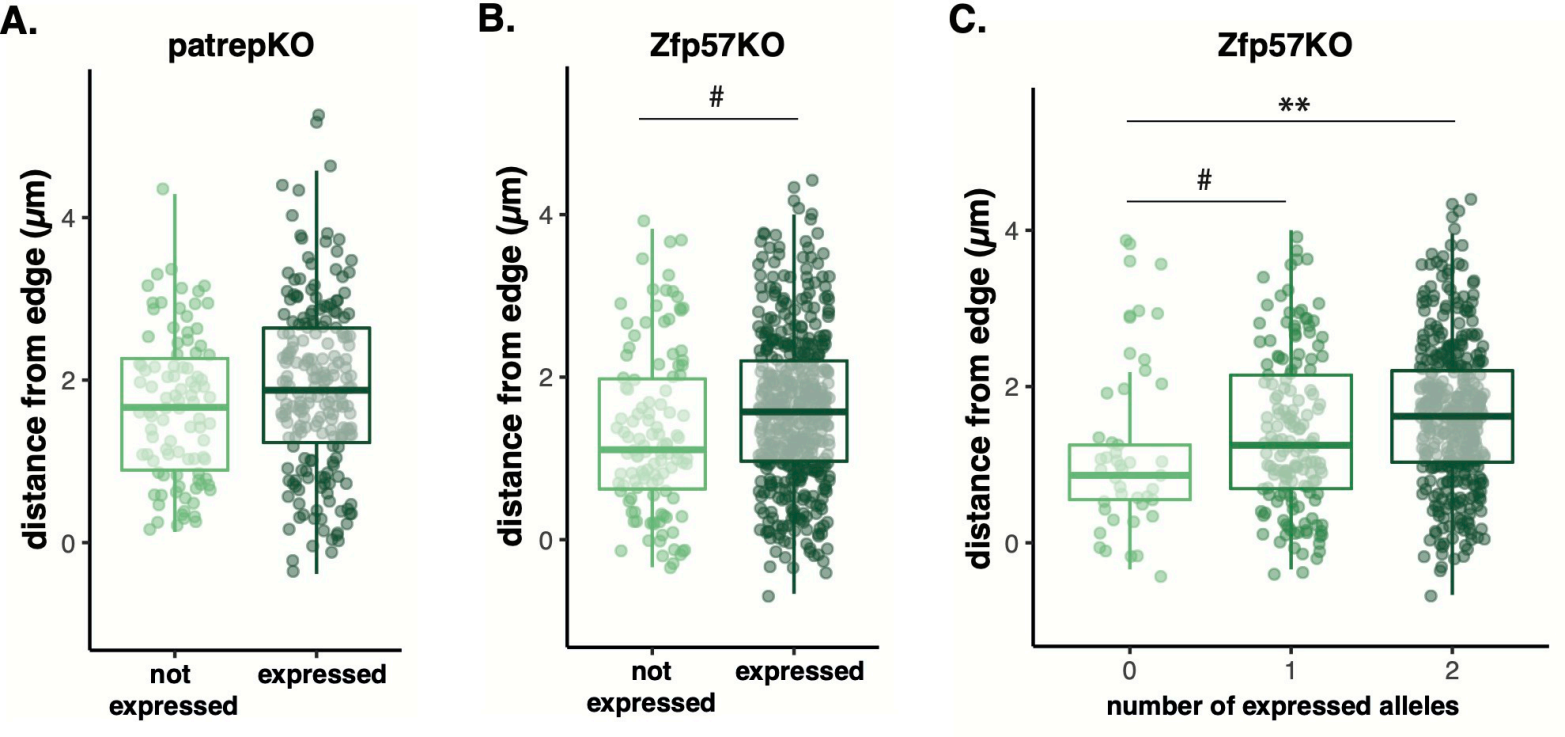
Distribution of alleles depending on *Gtl2/Meg3* gene expression state. (A) There was no relationship between localisation (location of the L1 probe relative to the periphery) and *Gtl2/Meg3* expression state from patrepKO ESCs. (B) Maternalized alleles from all Zfp57KO ES cells were marginally closer to the edge when *Gtl2/Meg3* was non-expressed (*#p<0*.*1*), (C) Maternal(ized) cells split by number of alleles that were expressed. Alleles in monoallelically expressed cells were marginally farther from the periphery than cells in which no alleles were expressed. However, cells that had both alleles expressed within the cell (biallelic expression) were significantly further away from the periphery *(#p<0*.*1*, *p<0.*05*).

While *Dlk1* is not expressed in ES cells, *Gtl2/Meg3* is strongly expressed. Though all maternally inherited alleles of the *Gtl2/Meg3* gene have the potential to express the gene, it is not active in all cells (S5 Fig). It is not known whether this heterogeneity in expression is stochastic or regulated. We know that, gene expression can be fine-tuned by transcription on-off cycles, sometimes called “bursting”, which could reflect genes moving in and out of transcription factories [3]. Alternatively, some individual ES cells may change their properties resulting in changes in gene expression that could include *Meg3* (*i*.*e*., through differentiation). Since we cannot distinguish these possible types of repression in our experiments, we refer to *Gtl2/Meg3*-non-expressing maternal alleles as “stochastic or developmental repression”. Stochastic or developmental repression of the canonically active maternal allele of *Gtl2/Meg3* was observed in 29.5% of WT ES cells and 32.3% of patRepKO ES cells (S5 Fig). We took advantage of this property to test whether *Gtl2/Meg3* alleles differed in their intranuclear localisation (*i*.*e*., distance from L1 to the nuclear envelope) based on their expression state as measured by RNA FISH (for the active alleles). We used a logistic regression approach to test if distance to the periphery predicted the expression state of *Gtl2/Meg3* (after controlling for nuclear volume variation). In three biological replicates each of patrepKO ES cell lines in which the single maternally inherited *Meg3* allele has the potential to be either expressed or not, we found no difference in localisation regardless of the expression of *Gtl2/Meg3* (z = 1.059, p = n.s; Fig 3A).

However, since Zfp57KO ES cells have twice as many maternal(ized) alleles as patrepKO ES cells, we reasoned that experiments with these cell lines would have greater power to examine the relationship between distance and expression. Using the same statistical methodology and same numbers of replicates, we found a non-significant trend (estimate = 0.927μm, p = 0.0721, Fig 3B) with non-expressed alleles being marginally closer to the periphery compared to expressed alleles. As before, nuclear volume was taken into account. Together these findings indicate insignificant effects of imprinted repression (*i*.*e*., epigenetic silencing) on localisation relative to the nuclear periphery. We, therefore, focused on maternalised expressed alleles to consider whether stochastic expression states rather than imprinted repression might better predict localisation.

### Gene expression predicts localisation better than parental epigenotype

Using Zfp57KO cells, we can measure whether biallelic expression could predict distance from the nuclear border better than monoallelic expression or no expression from either allele. Using a multiple linear mixed model approach we find that there is a marginal increase in distance when only one allele within the cell is expressed (t = 1.837, p = 0.067, estimate (slope) = 0.175 μm) but alleles move significantly further from the nuclear border when both alleles are expressed (t = 1.989, p<0.05, estimate (slope) = 0.185 μm; Fig 3C). This suggests that taking into account transcriptional state predicts a more centralised localisation. This could be due to gene dosage or reflect the overall increase in transcriptional activity of two active alleles within a cell. Nevertheless, it suggests that activation is correlated with a shift away from the nuclear border. This is consistent with the idea that maternalised repressed alleles are more similar in localisation to paternal alleles (epigenetically silenced) than paternal alleles that have escaped imprinting (S6 Fig).

**Fig 4:**
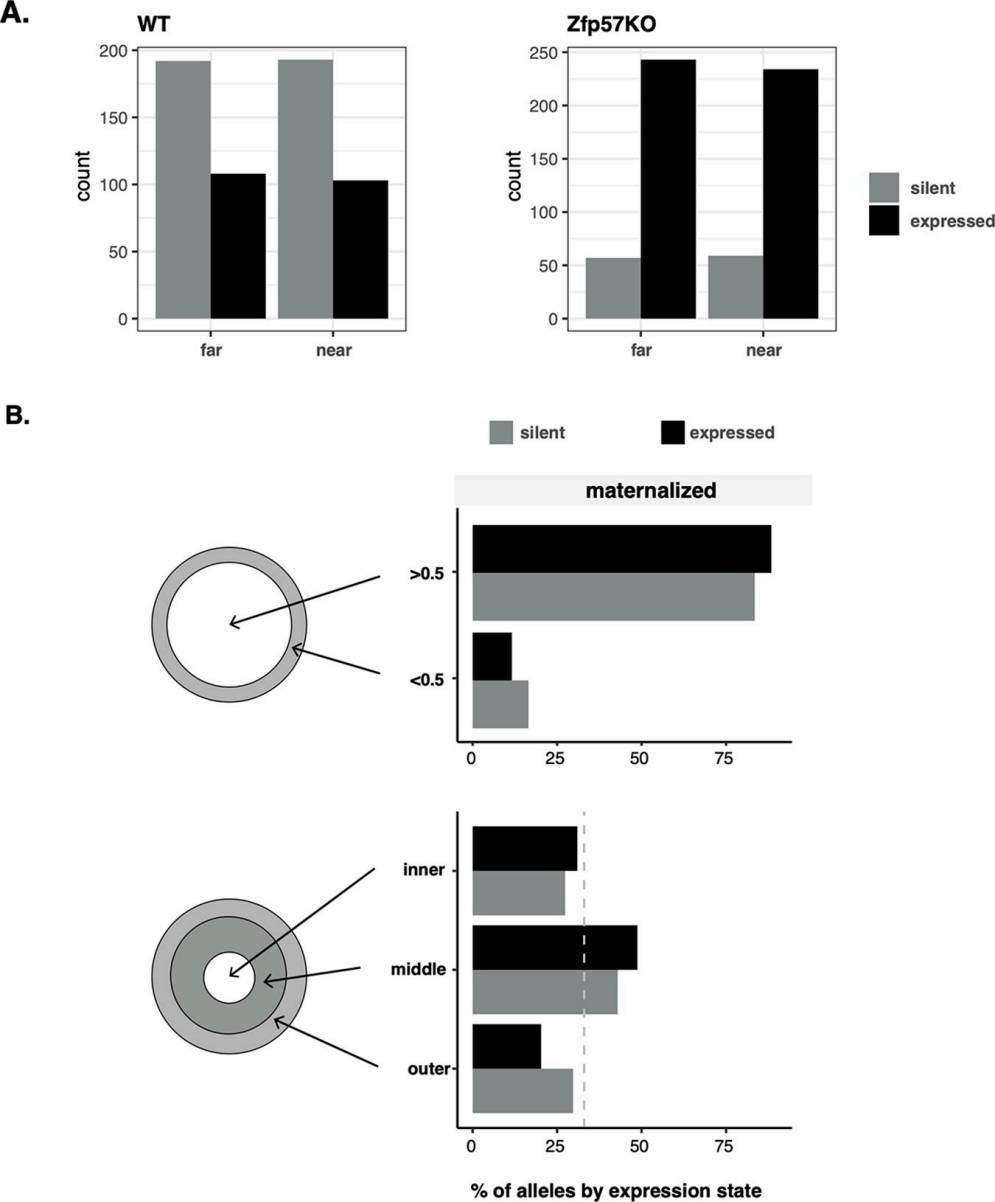
*Gtl2/Meg3*-non-expressing maternal(ized) alleles are not enriched at the nuclear periphery. (A) Being the near vs far allele did not affect the likelihood of being expressed in either wild-type (WT) or Zfp57KO ES cells. (B) The majority of maternal (patrepKO) and maternalised alleles (Zfp57KO) are localised away from the periphery with no difference between *Gtl2/Meg3* expressing and non-expressing alleles (top). Nor were there any significant differences in distribution of expressed and non-expressed alleles when the nucleus was divided into three equal volume bins (an inner, middle and outer; bottom).

We further explored the relationship between localisation and expression by examining whether alleles classified as ‘near’ and ‘far’ from the nuclear border were more or less likely to be expressed based on their relative position. We find no such relationship in WT (log_2_(OR) = 0.95, *p* = 0.80; Fishers Test) or Zfp57KO cells (log_2_(OR) = 0.93, *p* = 0.77; Fishers Test; Fig 4A) suggesting that both near and far alleles have an equal probability of being expressed. This does not consider distance from the periphery but rather the relative position of the two alleles within the nucleus. Hence, this makes no claims about the function of the nuclear periphery since it is possible that both maternal and paternal alleles in this model are too far from the periphery to undergo lamina-associated repression. Consistent with this and as shown in Fig 4B, repressed maternalised alleles (patrepKO and Zfp57KO combined here) are not more likely to be greater than 0.5μm away from the periphery (***χ***^**2**^ = 0.10, df = 1, p = n.s). To examine this at higher resolution, we plotted the regional location after dividing the nucleus into thirds by volume (outer, middle, inner) as has been described previously [20]. Results suggest that stochastically repressed alleles are not preferentially located to the periphery (***χ***^**2**^ = 2.3834, df = 2, p = n.s; Fig 4B). A similar pattern was found in WT cells (see S7 Fig), indicating that there is no distinct areas of localisation between expressed and transcriptionally silent alleles.

## Discussion

We performed 3D RNA-DNA FISH measurements in a total of 18 mouse ES cell lines and observed a very small but overall highly significant effect for *Gtl2/Meg3*-expressing alleles of the *Dlk1-Dio3* imprinted region to be localized towards the nuclear interior and for non-expressing alleles to be localized towards the nuclear periphery. As has been shown before [20], paternal alleles, which do not express *Gtl2/Meg3*, localize overall closer to the periphery than maternal alleles, from which *Gtl2/Meg3* is expressed in 70.5% of WT ES cells. Differences in localisation between paternalized and maternalized alleles (*i*.*e*., IGDMRKO *vs* ZFP57KO) are only evident when expression state is considered. In other words, expressed *Gtl2/Meg3* alleles are positioned further away from the periphery than non-expressed alleles. This idea is consistent with previous work showing that the tethering of transcriptional activators to a peripheral chromosome site can lead to movement of the chromosome site towards the nuclear interior [31,32]. Furthermore, the position within the nucleus is directly related to the number of alleles being expressed within a given cell, as evident through the analysis of cells expressing zero, one or two alleles. Future studies can address the biological relevance, if any, of this intriguing finding.

The effect size of the localisation difference between expressing and non-expressing alleles in our study is much smaller than that previously described (19). Three notable differences between our work and the previous study might explain this: 1) Kota et al. [33] used *Gtl2/Meg3* expression as a read-out for parental origin while we included an independent DNA marker and genetic approaches allowing us to distinguish and quantify expressing and non-expressing maternal chromosomes. 2) Kota et al. [33] used fewer biological replicates hence would not have been able to take into account the variability that is evident between lines. 3) Our measurements utilise a DNA marker located 400kb away from the interrogated gene which might result in lower data resolution (S1 Fig). Both genetic locations however, lie within the same TAD in ES cells [24,25] and show frequent overlap in a double probe DNA FISH experiment using probes against the L1 repeat and the *Gtl2/Meg3* gene (S1 and S8 Figs). However, these locations do lie in different sub-TADs which may partially explain discrepancies between our results and Kota et al. [33]. Our findings reflect the importance of independently distinguishing the parental chromosomes themselves from their expression, and then determining the contribution that each makes to subnuclear localisation.

Mouse ES cells show an unusual cell cycle distribution where a majority of cells (75%) will be in S-phase in a growing cell population [34]. S-phase can potentially have an influence on gene expression and cell cycle heterogeneity could thus be a source of variability between biological replicates. However, when comparing nuclear sizes (as a proxy for cell-cycle differences) of expressing and non-expressing alleles as well as biological replicates, some small variation in nuclear size was evident in some instances (S9 Fig). We therefore included nuclear volume as a covariate in all of our analyses to correct for any minor fluctuations in nuclear volume. Hence, all of our estimates of the differences in allelic distribution control for minor changes in nuclear volume.

Though data from all our ES cell lines show a significant expression-dependent effect on subnuclear localisation, the effect size is extremely small with *Gtl2/Meg3*-non-expressing alleles being on average only 170 nm closer to the nuclear periphery than *Gtl2/Meg3*-expressing alleles. Indeed, most expressed alleles can be more broadly categorised as being away from the periphery (or outer most region) though not extremely central to the nucleus. It is interesting to consider whether such a small effect is biologically meaningful. Since we did not observe a significant enrichment of inactive alleles in the immediate vicinity of the nuclear envelope, inactivating interactions with the nuclear lamina are unlikely to play a major functional role at this imprinted domain. Notably, *Gtl2/Meg3* expression was regularly found right at the edge of DAPI staining probably representing areas of low-density chromatin at the very periphery of nuclei (see methods section for details). Although it can be envisioned that in some cases *Gtl2/Meg3* expression might have been inhibited by transient nuclear envelope interactions and had already moved away from the periphery at the time of the experiment, such alleles should still show up as peripheral enrichment since mobility of individual loci during interphase is usually limited to about 1 μm and it has been shown that large scale changes in localization require cell division [1,35].

A more likely explanation is that the observed pattern of allele localisation is due to some activating effect of structures at the nuclear interior. This is consistent with the increase in internalisation observed when two alleles are expressed compared to one in a given cell. Transcription is known to happen preferentially at sites where the transcriptional machinery and active genes are clustered into so-called transcription factories and adjacent sites known as speckles [3,4,32]. Such factories are found in open chromatin at the borders of chromosomal territories and it seems logical that they would be biased to be more frequent at the more transcriptionally favourable nuclear interior. Therefore, one possible explanation for the observed non-enrichment at the nuclear periphery and the small effect size of our expression-localisation correlation could be that *Gtl2/Meg3*-non-expressing alleles are evenly distributed within the space they can take up around the chromosome territory, independent of nuclear envelope interactions, while *Gtl2/Meg3*-expressing alleles need to be localized at transcription factories which are biased to be at the interior. It would be interesting to test this idea by co-localising expressing and non-expressing alleles with components of transcriptional factories. Nevertheless, our use of multiple biological replicates in multiple genetic models highlights the wide range of positions that an allele can take within the nucleus regardless of expression status.

For all correlations of nuclear localisation and expression, the question of cause and consequence arises. Is peripheral localisation used as a means to fine-tune gene expression by making the co-localisation with transcription factories more or less likely? The absence of enrichment of inactive alleles at the nuclear periphery does not argue for such a mechanism. Kota and colleagues have shown that position changes of the *Dlk1-Dio3* region are local and do not involve gross changes in chromosome territory [20]. Similarly, our Zfp57 mutants show that *cis* acting epigenetic changes relate to a shift in localisation at the same time as inducing *Gtl2/Meg3* gene expression. This argues that a nuclear envelope anchoring mechanism acting at a distance is unlikely to be involved. A local anchoring mechanism should show up as peripheral enrichment and we do not observe this. We therefore suggest that the small shift in peripheral localisation that we observe is a consequence, rather than a cause of expression changes and that the more functionally relevant aspect is the interior location of expressed alleles.

## Methods

### Cell culture

Mutant and corresponding control ES cells were generated from single blastocyst using feeder-free-based 2i LIF culture conditions (N2B27, Stem Cell Sciences) [36]. In brief, morula embryos were collected from pregnant female mice and cultured in KSOM with 2i inhibitors, and then each blastocyst was genotyped using trophectoderm after immunosurgery. *del*^L1rep^ mice have an *albino* C57BL/6J background (BL6) [21] and were crossed to *albino* BL6 mice to obtain WT, maternal and paternal heterozygotes. IG-DMR heterozygous females [30] were mated with BL6 males to obtain IG-DMR maternal KO and control WT morula embryos. *Zfp57* zygotic KO ES cells and corresponding control ES cells were generated in a previous study [26]. We observed unexpectedly low expression levels of *Gtl2/Meg3* in two of five *Zfp57*KOES cell lines and therefore excluded these two cell lines from these studies. For our analysis of ES cells of all genotypes, we used data from the three biological replicates that showed the expected and similar proportions of non-expressing, monoallelically expressing, and biallelically expressing cells (S5 Fig). This research was approved by the Animal Welfare and Ethical Review Body (AWERB) of the University of Cambridge and conducted in accordance with UK Home Office Animals Scientific Procedures Act under project licence 80/2567.

### Fluorescence in situ hybridisation

We generated fluorescent probes for detection of the LINE repeat (BAC bMQ-177C10, obtained from CHORI) and nascent *Gtl2/Meg3* transcripts (fosmid WIBR1-2686H19, a kind gift from the Heard lab, Paris) by first amplifying plasmid mini preps with the Illustra TempliPhi Large Construct Kit (GE Healthcare) and subsequently labelling 2 μg of the amplification product by nick translation using Green UTP or Red UTP (Abbott Molecular) according to the manufacturer’s instructions. Sequential RNA and DNA 3D fluorescence in situ hybridisation (FISH) was performed as previously described [37]. However, ES cells were grown on laminin-coated coverslips prior to fixing to ensure well-spread monolayer growth and nuclei were counterstained with 0.2 μg/ml DAPI after hybridisation and washes. 3D image stacks of 10–15 positions per coverslip were acquired using a Carl Zeiss Axiovert 200M microscope with a 63x/1.25 Plan Apochromat objective taking 100 image planes per image stack at a z spacing of 0.15 μm. For double probe DNA FISH the same protocol was used as in sequential FISH and the Gtl2/Meg3 fosmid probe was used to detect the gene rather than nascent RNA.

### Image analysis

DNA FISH image stacks were post-processed using Huygens Professional deconvolution software (version 14.10.1p8, SVI, The Netherlands) and 3D measurements were taken using Fiji [38]. We developed a Fiji script that enabled us to generate binary representations of DAPI-stained nuclei and FISH signals and to take automated 3D measurements of and between these objects calling Fiji’s 3D manager function (Fig 1C). If FISH signals were duplicated due to DNA replication, we used the mean of both measurements for the analysis. Due to the nature of the binarization process using DAPI staining of chromatin, binary images of nuclei sometimes differed slightly in shape from what was seen by eye in the original image because, depending on image background levels or closeness of neighbouring nuclei, non-dense chromatin regions were interpreted as background by the algorithm. This led to a minority of DNA FISH signals (2.8%) being outside the nuclear volume. To avoid bias against peripherally located signals, we included such signals as negative distance measurements. In very rare cases the algorithm generated a bay-like background area around a FISH signal in a non-dense chromatin area, in a way that our standard automated measurements would lead to false results. Again, to avoid biasing against peripheral signals as well as inaccurate manual measuring, in these rare cases (0.3%) we used an alternative algorithm that approximates the nuclear volume by fitting an ellipse around the original shape and used this shape for distance measurements. For relative distance measurements, we used the “radiusCen” measurement of Fiji’s 3D manager, which measured the distance between the centre of the binary representation of the nucleus and its border through the centre of the binary representation of the FISH signal, as local nuclear radius. Relative distances were calculated as absolute distance / local nuclear radius. In parallel to DNA FISH analysis, the expression state of the *Gtl2/Meg3* gene was evaluated from original (non-deconvolved) nascent RNA FISH image stacks for each allele as either expressed (FISH signal above background level) or non-expressed (no signal).

### Lamin staining by immunofluorescence

E14tg2a ES cells were cultured on laminin-coated coverslips, fixed for 10 minutes in 3% paraformaldehyde and permeabilized in 0.5% Triton X-100. Cells were then blocked in 1% BSA for 15 minutes and then incubated in 1:200 Goat α Lamin B_1_ (Santa Cruz SC-30264) 1% BSA for 45 minutes at room temperature. The secondary antibody, 1:500 Donkey α goat IgG Alexa 633 1% BSA, was applied and cells incubated for 40 minutes at room temperature in a dark humid chamber. The cells were then counterstained for 1 minute in 1X PBS containing 0.2mg/ml DAPI and mounted in mounting medium. 3D image stacks were acquired (see Fluorescence in situ hybridisation methods) and image stacks were post-processed using Huygens Professional deconvolution 338 software (version 14.10.1p8, SVI, The Netherlands). Distances were measured using Fiji.

### Statistical methodology

All analyses used a linear regression model framework using nuclear size as a covariate (to control for any volume effects on expression and/or localisation) using base R regression functions [39]. Given that some cells had measurements for multiple alleles, where appropriate, we used mixed effects multiple linear regression models using the lme4 and lmerTest packages in R [40,41] to allow for random intercepts (of unique cells) which effectively controls for variability arising from the repeated measurement of unique cells [42]. Data handling and visualisation in R was carried out using the ‘tidyverse’ packages [43]. Chi-squared tests for differences in nuclear volume distributions were calculated using base chi.sq functions in R. Fishers’ tests of enrichment were performed using the base R fisher.test function.

## Supporting information

S1 Fig UCSC (https://genome.ucsc.edu/) -screenshot of the Dlk1-Dio3 imprinted region in mouse mm9 showing the exact positions of RNA (Meg3) and DNA (L1 repeat) FISH probes, as well as the genomic distances between them (inner, central and outer distance). The exact position of the LINE1 repeat deletion [21] in relation to the repeat probe and the UCSC RepeatMasker are also shown. The top panel shows ES cell TADs from Schoenfelder and colleagues (Bab_ESC) and Dixon and colleagues (Ren_ESC) loaded as custom tracks [24,25]
(TIFF)

S2 Fig Correlation plots of DAPI edge measurements (μm) compared to lamin inner (a) and outer (b) borders (μm). (TIFF)

S3 Fig Plots of the distance from the nuclear border relative to *Gtl2/Meg3* expression in individual biological replicates of all KO and WT groups measured in the study. We find no significant effect of replicate on overall distance measures.
(TIFF)

S4 Fig Additional examples of gene expression in the five ES cells genotypes at larger magnifications (compare Fig 1). *Gtl2/Meg3* probe stained images are from nascent RNA FISH and L1 Repeat probe stained images are from subsequent DNA FISH. The FISH images represent maximum projections of 10–30 central z planes of acquired image stacks. Blow-ups of framed regions are shown on the right. Scale bars represent 5 μm.
(TIFF)

S5 Fig Numbers of cells per cell line with no, monoallelic, or biallelic *Gtl2/Meg3* expression. (TIFF)

S6 Fig A) Distribution of alleles split by genotype and expression-type. Non-expressed maternal and maternalised alleles look similar in distribution to unexpressed paternal and paternalised alleles (not significantly different). Activated alleles (either through imprinting escape or normal maternalised expression) are generally further away from the nuclear border. We did formally test the effect of imprinting escape because of low power in this group.
(TIFF)

S7 Fig A) The majority of maternalised alleles (patrepKO and Zfp57KO) are localised away from the periphery with no difference between *Gtl2/Meg3* expressing and non-expressing alleles (data presented as a percentage of total alleles). B) Nor were there any significant differences in distribution of expressed and non-expressed alleles when the nucleus was divided into three equal volume bins (an inner, middle and outer; bottom) C) This was similar to WT cells, presented both as C) a proportion of all alleles and D) a proportion of expression state (comparable to Fig 4A in the main text).
(TIFF)

S8 Fig Double probe DNA FISH in WT ES cells (ES cell line WT8). The images show only one z image plane. All three locations, at which the L1 repeat signal and the *Gtl2/Meg2* gene signal have their centres roughly in the same z image plane, are shown as blow-ups on the right. The distance between the signal centres is between 0.25 and 0.36 μm in these three examples.
(TIFF)

S9 Fig The density of nuclear sizes does not vary considerably between *Gtl2/Meg3*-expressing and -non-expressing ES cells and therefore does not hint towards a specific down- or upregulation of gene activity during s-phase. (TIFF)

S1 Table CSV file of raw data and metadata from all experimental cell lines. (CSV)

S1 Code RMarkdown file with code for analysis. (RMD)
