# Supplementary figures and images for: Subnuclear localisation is associated with gene expression more than parental origin at the imprinted Dlk1-Dio3 locus
Source: PLoS Genet. 2022 Apr 28;18(4):e1010186. doi: 10.1371/journal.pgen.1010186 (PMC9129038; doi:10.1371/journal.pgen.1010186)

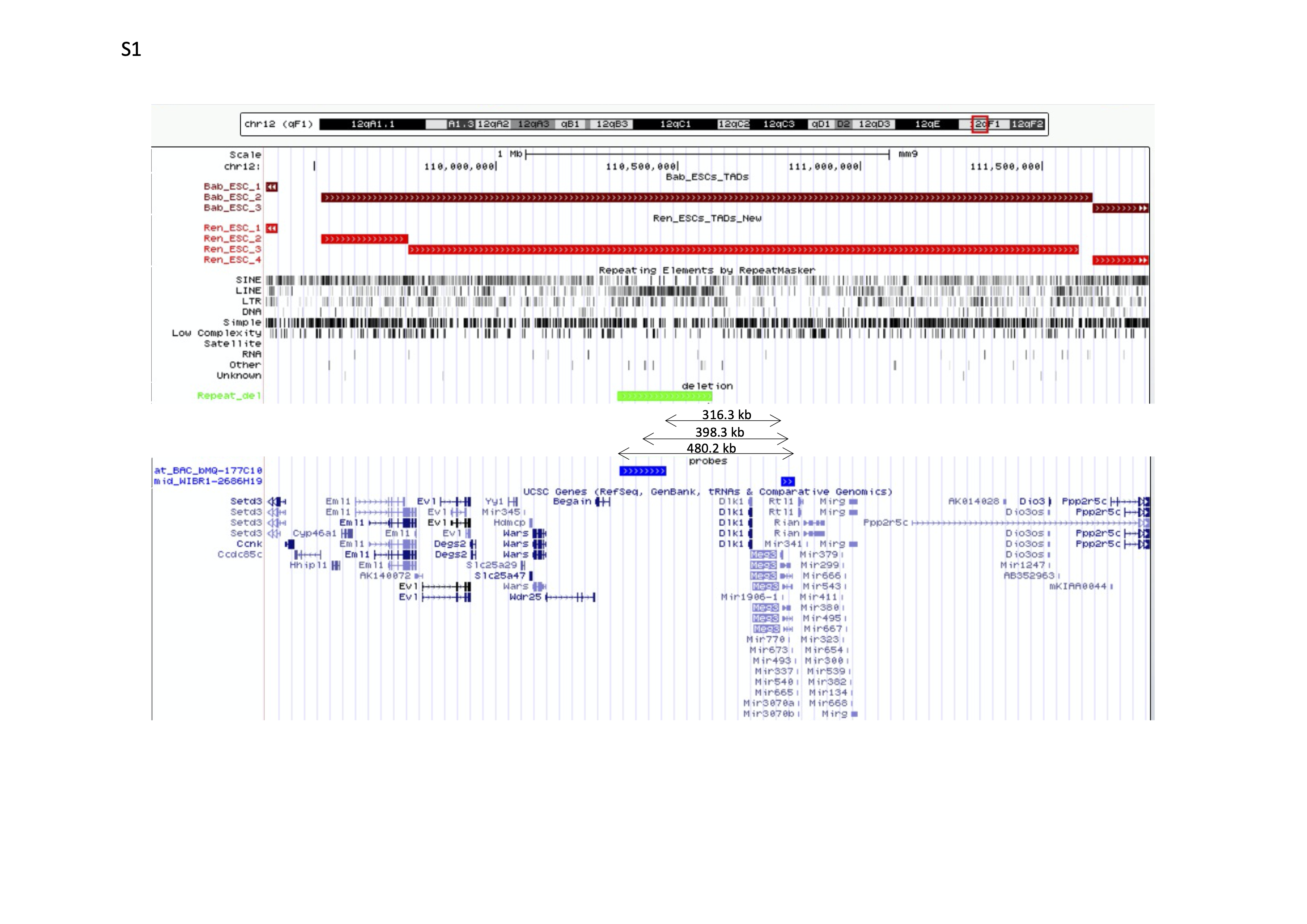

Supplement: S1 Fig — The exact position of the LINE1 repeat deletion [21] in relation to the repeat probe and the UCSC RepeatMasker are also shown. The top panel shows ES cell TADs from Schoenfelder and colleagues (Bab_ESC) and Dixon and colleagues (Ren_ESC) loaded as custom tracks [24,25] (TIFF) [file pgen.1010186.s001.tiff]

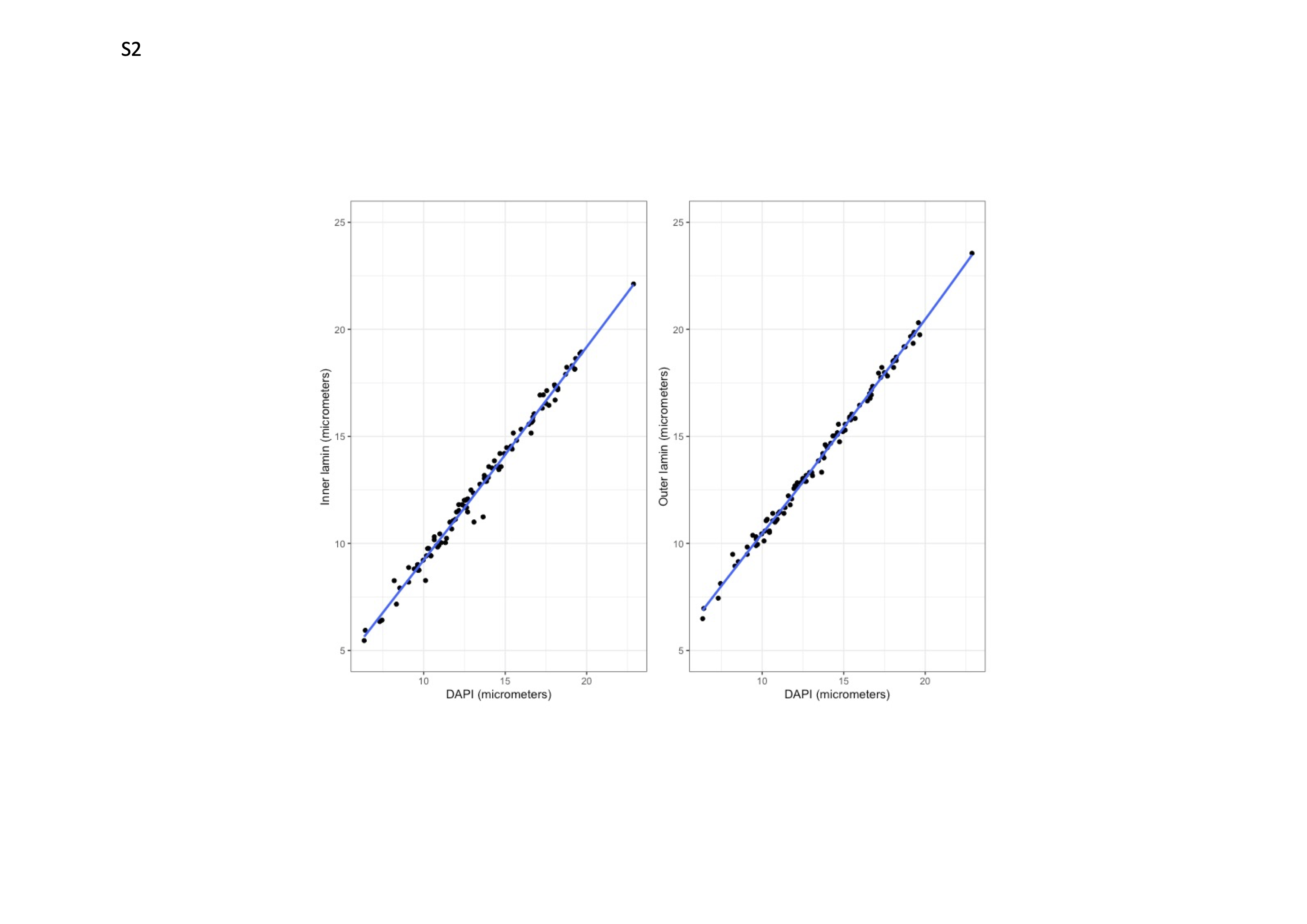

Supplement: S2 Fig — (TIFF) [file pgen.1010186.s002.tiff]

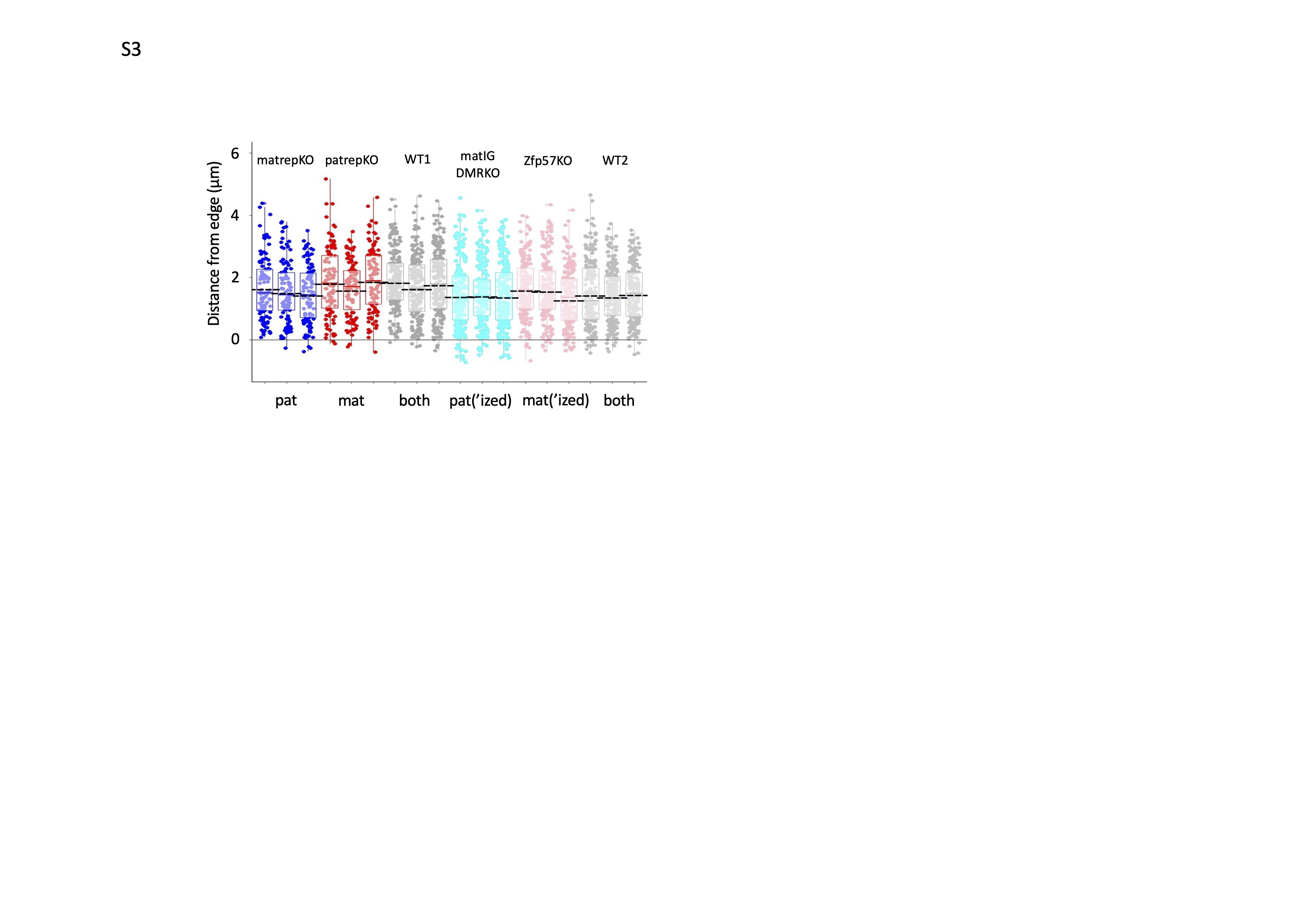

Supplement: S3 Fig — We find no significant effect of replicate on overall distance measures. (TIFF) [file pgen.1010186.s003.tiff]

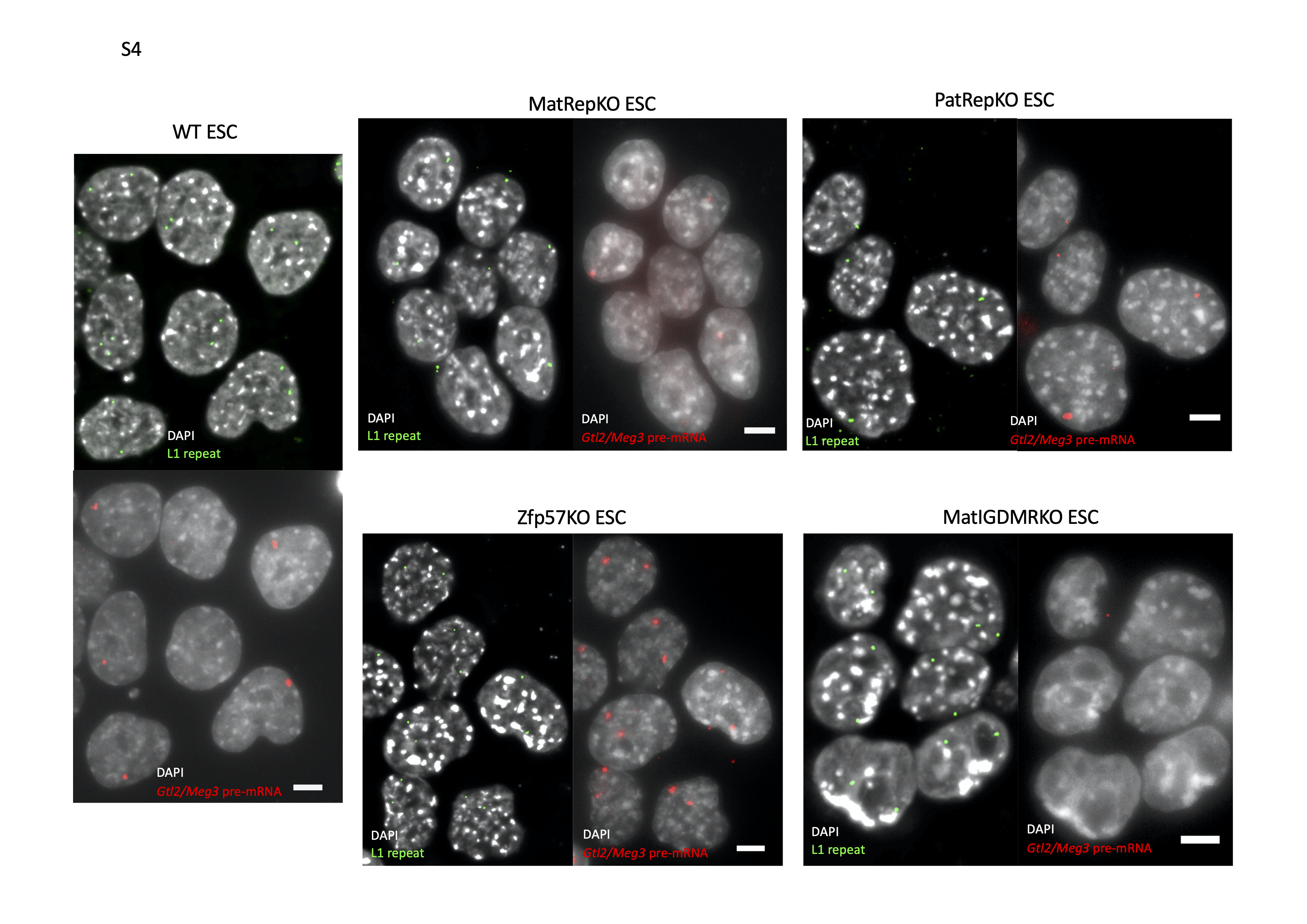

Supplement: S4 Fig — Gtl2/Meg3 probe stained images are from nascent RNA FISH and L1 Repeat probe stained images are from subsequent DNA FISH. The FISH images represent maximum projections of 10–30 central z planes of acquired image stacks. Blow-ups of framed regions are shown on the right. Scale bars represent 5 μm. (TIFF) [file pgen.1010186.s004.tiff]

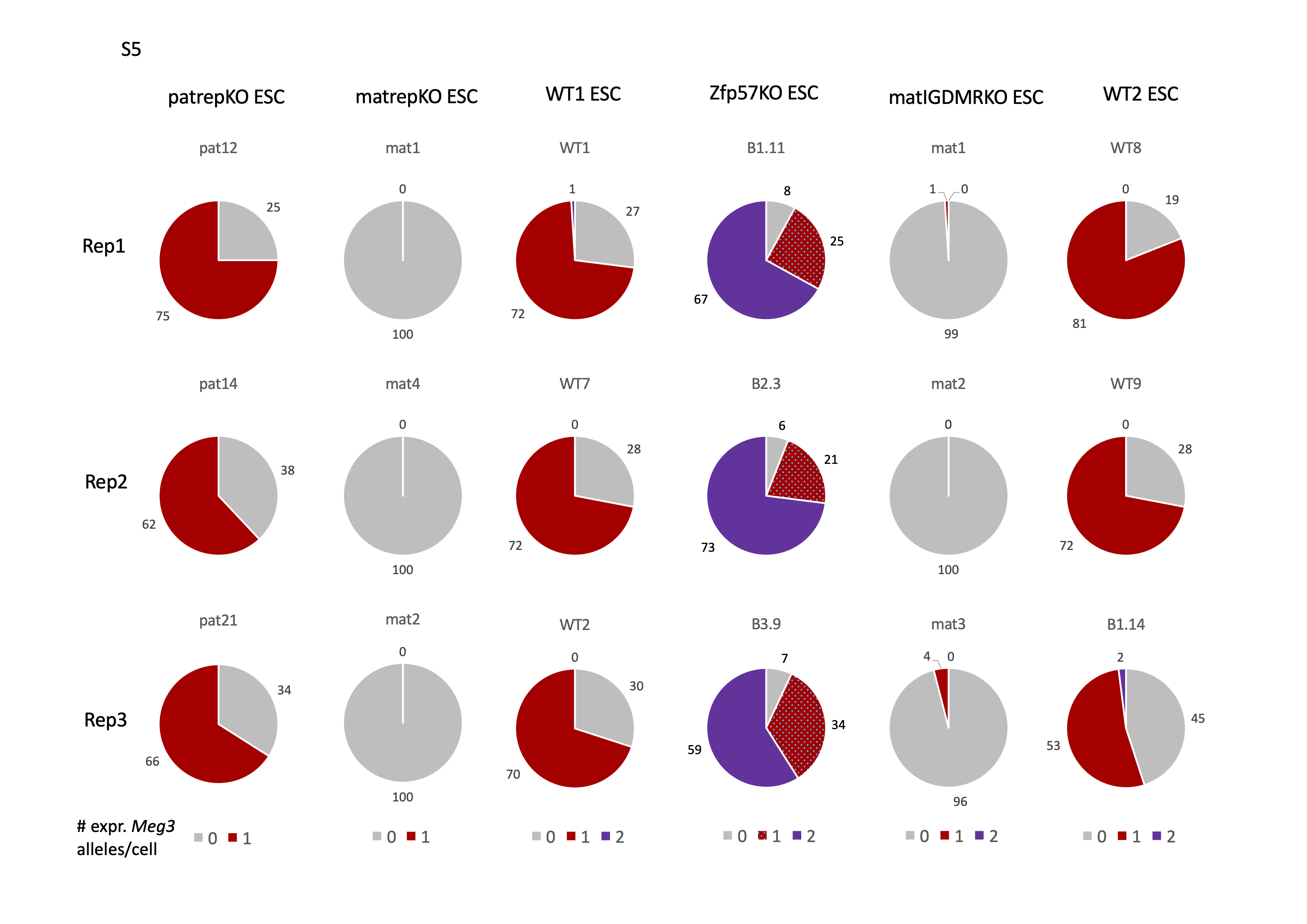

Supplement: S5 Fig — (TIFF) [file pgen.1010186.s005.tiff]

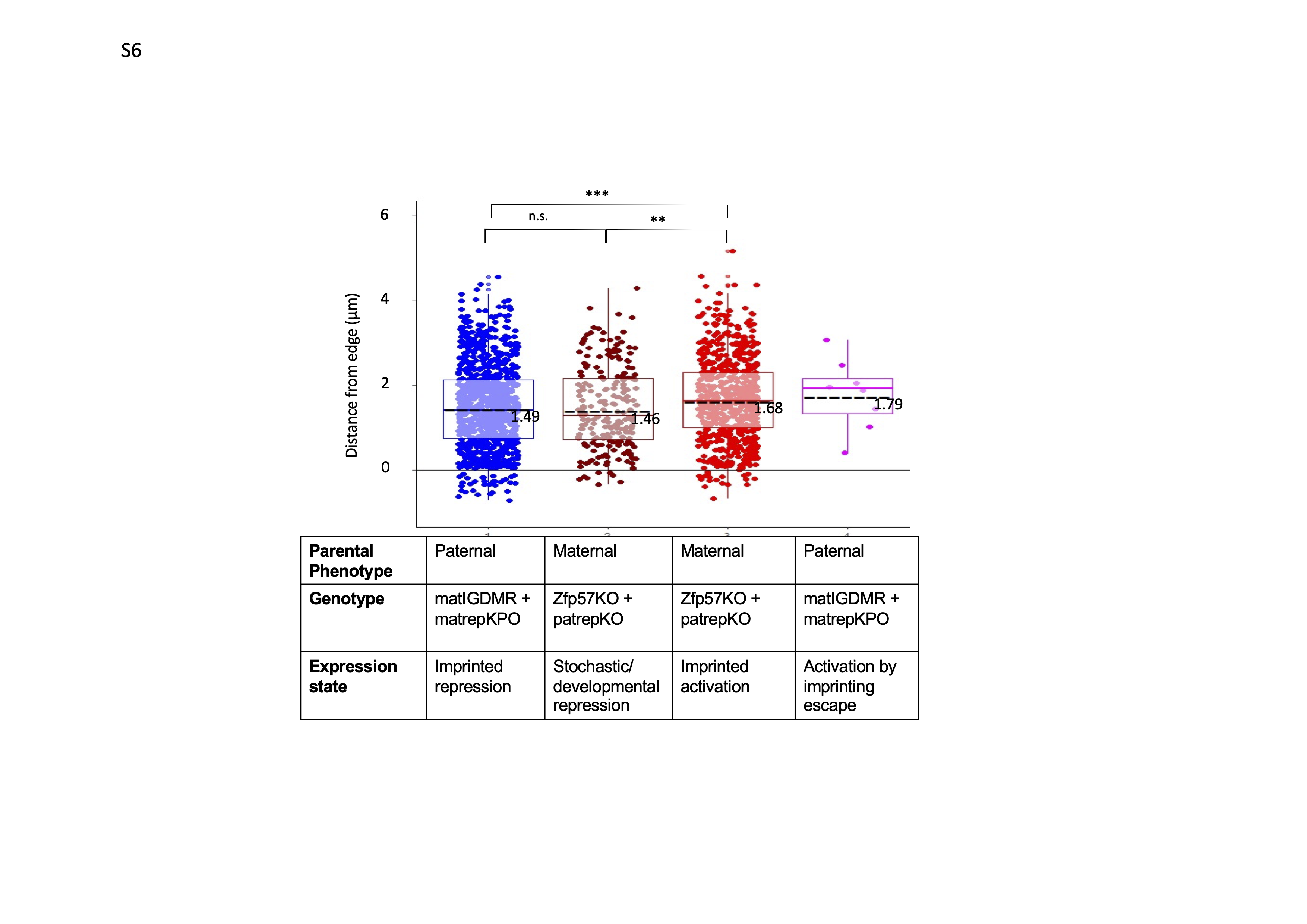

Supplement: S6 Fig — A) Distribution of alleles split by genotype and expression-type. Non-expressed maternal and maternalised alleles look similar in distribution to unexpressed paternal and paternalised alleles (not significantly different). Activated alleles (either through imprinting escape or normal maternalised expression) are generally further away from the nuclear border. We did formally test the effect of imprinting escape because of low power in this group. (TIFF) [file pgen.1010186.s006.tiff]

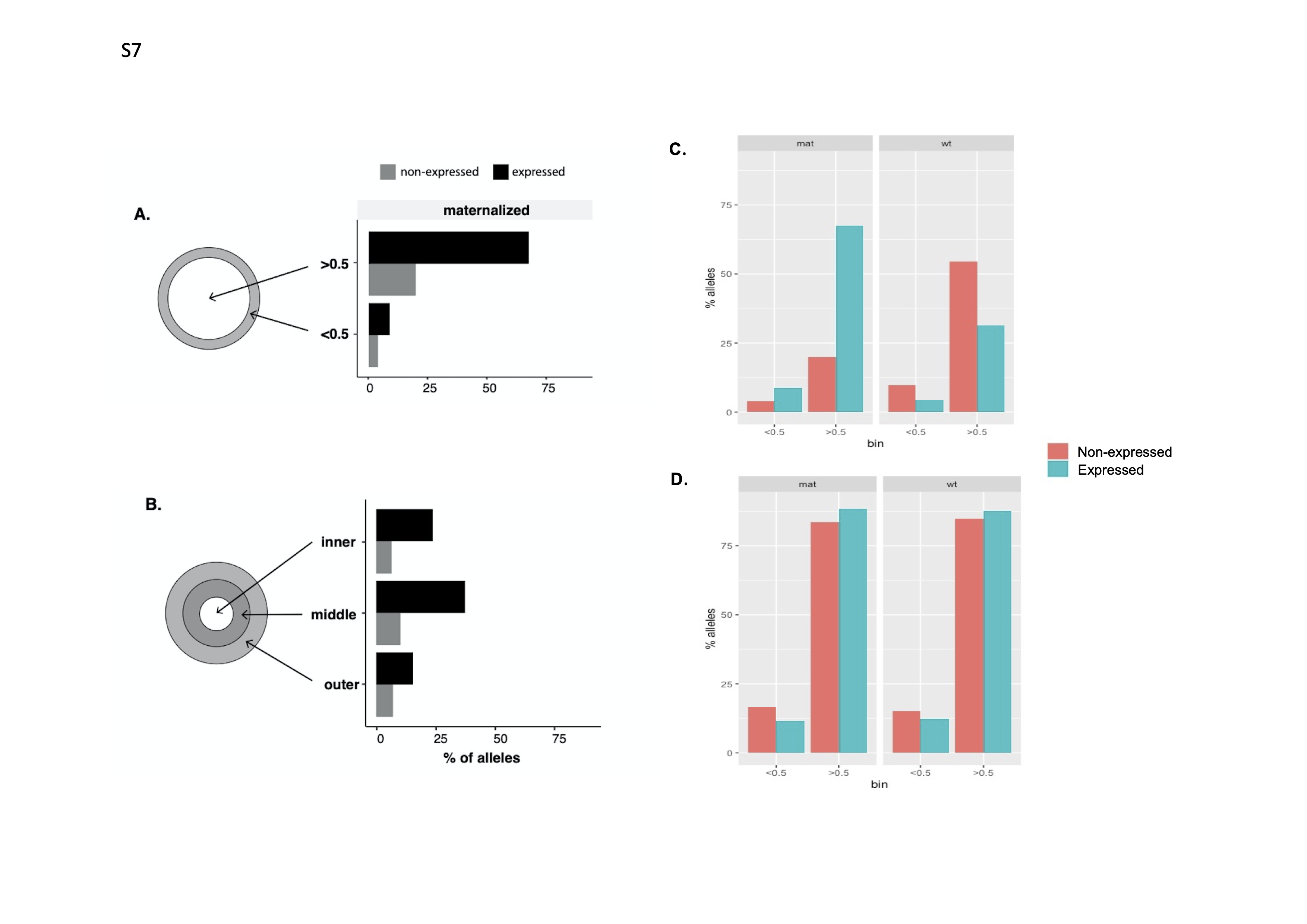

Supplement: S7 Fig — A) The majority of maternalised alleles (patrepKO and Zfp57KO) are localised away from the periphery with no difference between Gtl2/Meg3 expressing and non-expressing alleles (data presented as a percentage of total alleles). B) Nor were there any significant differences in distribution of expressed and non-expressed alleles when the nucleus was divided into three equal volume bins (an inner, middle and outer; bottom) C) This was similar to WT cells, presented both as C) a proportion of all alleles and D) a proportion of expression state (comparable to Fig 4A in the main text). (TIFF) [file pgen.1010186.s007.tiff]

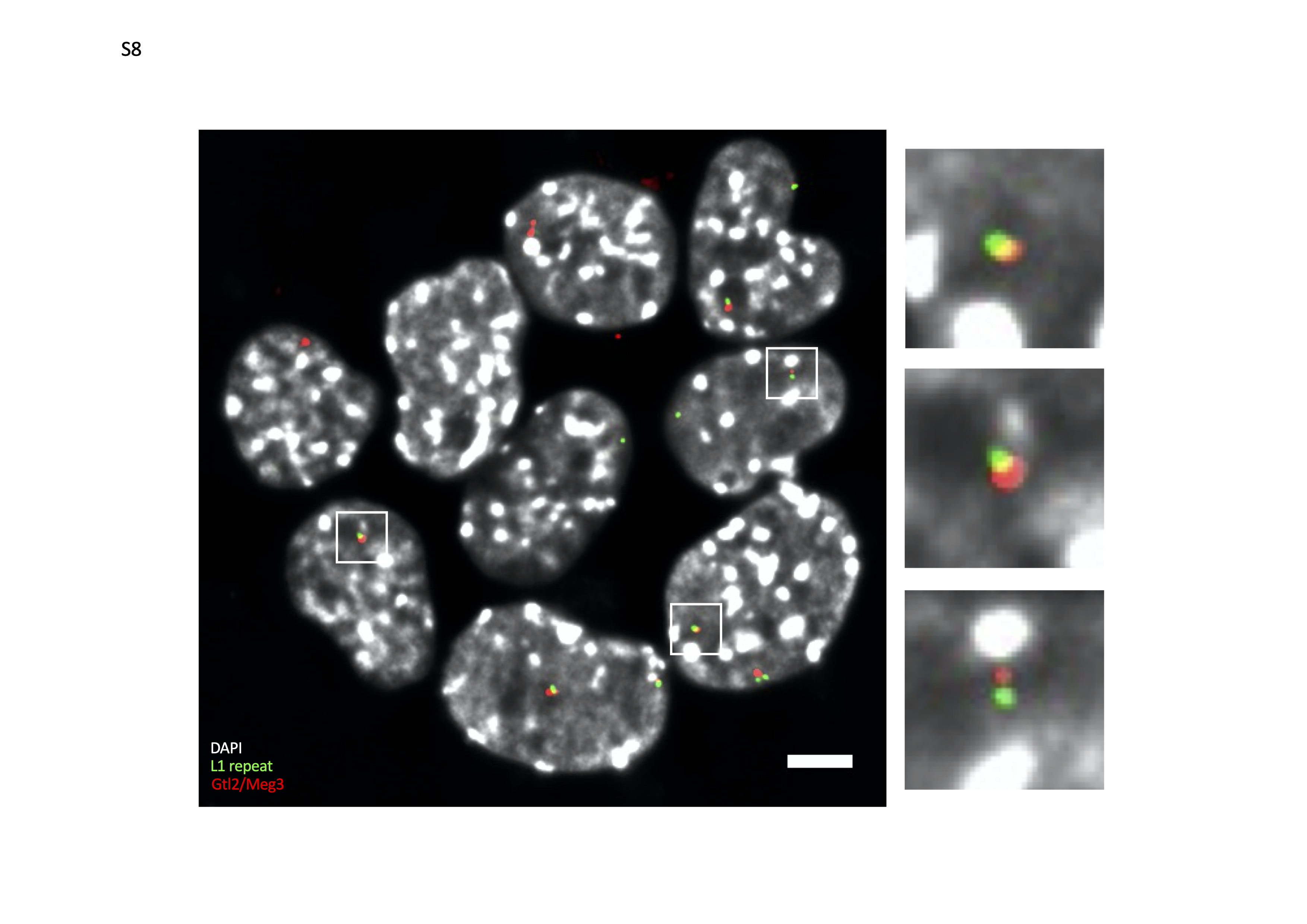

Supplement: S8 Fig — The images show only one z image plane. All three locations, at which the L1 repeat signal and the Gtl2/Meg2 gene signal have their centres roughly in the same z image plane, are shown as blow-ups on the right. The distance between the signal centres is between 0.25 and 0.36 μm in these three examples. (TIFF) [file pgen.1010186.s008.tiff]

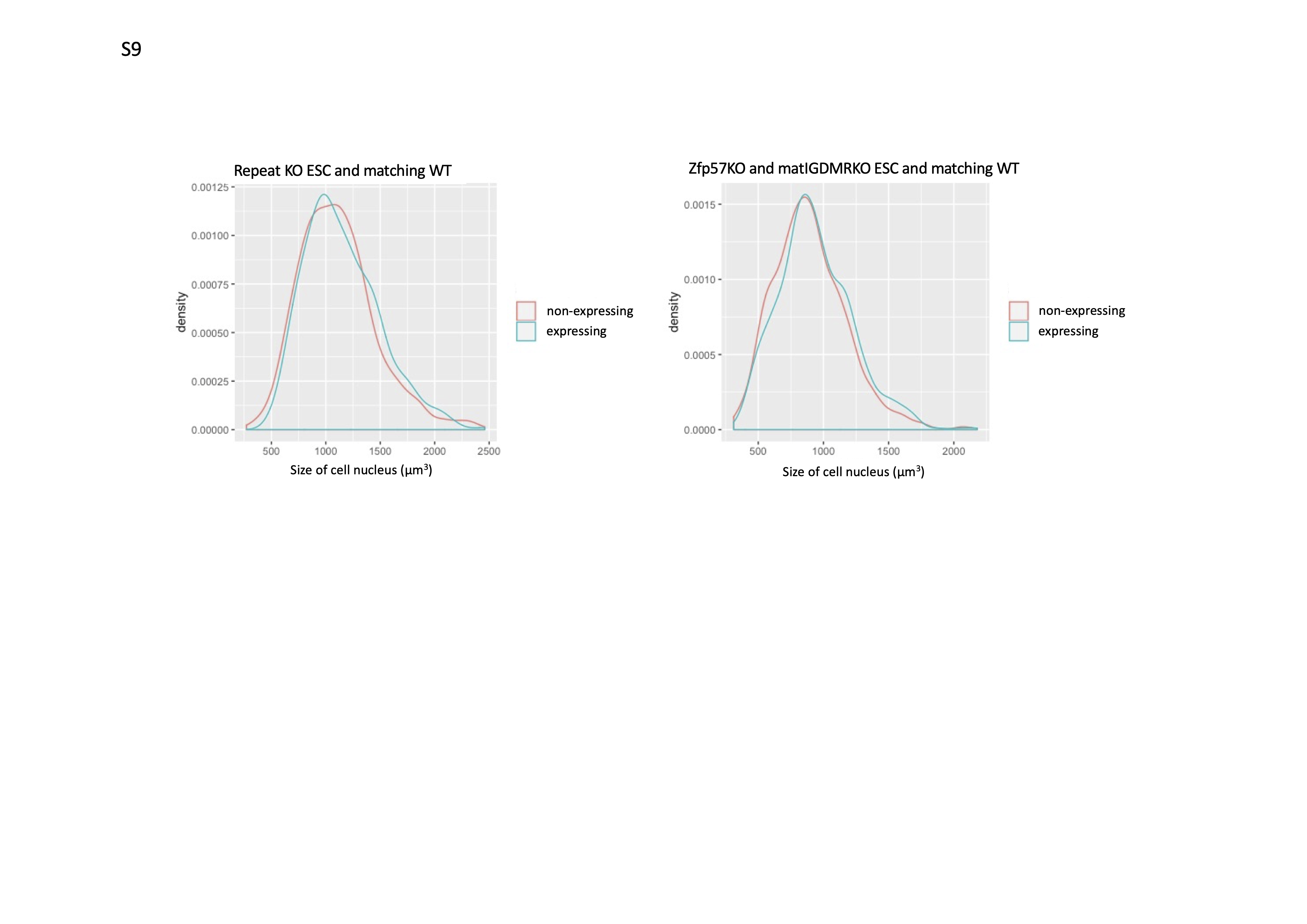

Supplement: S9 Fig — (TIFF) [file pgen.1010186.s009.tiff]
